# Psychopathology and psychotherapeutic intervention in diabetes: particularities, challenges, and limits

**Published:** 2016

**Authors:** O Popa-Velea, L Bubulac, L Petrescu, RM Purcarea

**Affiliations:** * Department of Medical Psychology, “Carol Davila” University of Medicine and Pharmacy, Bucharest, Romania; ** Department of Internal Medicine, “Dr. Carol Davila” Clinical Nephrology Hospital, Bucharest, Romania

**Keywords:** diabetes, psychological, psychotherapy

## Abstract

Diabetes is a problem of great public health importance, creating a considerable burden to the affected individuals and society. The psychological approach of this disease implies the early acknowledging of behavioral symptoms and the construction of effective psychotherapeutic interventions.

Regarding the psychological symptoms, cognitive malfunctions in diabetes include a slowing of information processing, attention, memory, and concentration, which, in turn, can significantly diminish motivation for therapy, compliance, and ability for self-care. Restrictions pertaining to daily activities, risks of treatment itself and the perceived inability to control the disease can furthermore reduce the perceived quality of life of these patients. Depression can complicate the picture, by a supplementary decrease in compliance and an increase of care expenses.

A proper management of diabetes involves a joint action of the patient, physician, and the psychologist. A better self-care can include commuting from passive to active coping, getting informed, maintaining realistic hopes, and long-term thinking. Physicians can express more consistent empathy, thereby increasing confidence. A substantial gain can be brought by considering variables involved in modulating compliance (e.g. the patient’s representations of gains and losses, group norms, ability vs. desire of control). Psychotherapeutic interventions include techniques such as counseling, cognitive-behavioral therapy, relaxation, hypnosis, and family therapy.

## Introduction

Diabetes is a problem of great public health importance, creating a considerable burden to the affected individuals and society. About 70,000 children worldwide are prone to develop type 1 diabetes every year, with an overall annual increase in incidence being estimated at around 3% [**1**].The introduction of new instruments of testing and monitoring diabetes has contributed, to a certain point, to a decrease in the mortality rates in the past decade. However, a further decrease of mortality and increase of quality of life can be expected from the intervention on additional factors involved in the pathogeny of diabetes, such as psychological variables. This paper represents an overview of the main psychological consequences of diabetes and of their management.

## Psychological consequences of diabetes

Comprises deteriorations at various levels, from the perception of the disease to frank psychiatric co-morbidity and a persistent decrease in self-reported quality of life.

## Cognitive malfunctions: 

Several mechanisms are commonly reported in literature for their occurrence:

1. hyperglycemic-induced end-organ damage (via reactive oxygen species) => neuronal damage and altered neurotransmitter function [**2**];

2. ischemia (diabetic microangiopathy) + hyperglycemia => diffuse brain degeneration [**3**,**4**];

3. decreased global rates of cerebral blood flow, correlated with the duration of the disease [**5**];

4. ischemia (diabetic microangiopathy) + hyperglycemia => accumulation of glutamate => neuronal damage [**6**];

5. absence of C-peptide [**7**];

6. hypoglycemic-induced neuronal damage (cortex, basal ganglia, hippocampus) [**8**].

The main cognitive symptoms are directly or indirectly responsible for a lower therapeutic compliance and for a possible subsequent deterioration of the therapeutic relationship (**Table 1**):

**Table 1:** Most frequent cognitive impairments in diabetes

Type 1 diabetes	Type 2 diabetes
Slowing of information processing	Decreases in psychomotor speed [**9**,**10**]
Decrease in psychomotor efficiency	Deficits of the executive function [**10**-**11**]
Low attention	Poor verbal memory [**12**] and working memory [**11**,**13**]
Low mental flexibility	Low processing speed [**12**]
Loss of inhibition and focus (in teenagers) [**14**]	Decrease of complex motor functioning [**10**]
Impaired working memory	Delayed recall [**15**,**16**]
	Impairment of verbal fluency [**10**,**17**]
	Impairment of attention [**18**]
	Impairment of visual retention [**19**]

Long diabetes duration and young age of the onset of diabetes are considered the strongest predictors of a poor cognitive processing [**20**].

Some studies claim that, even before diabetes meets the clinical criteria, impaired glucose tolerance can be a risk for cognitive dysfunction itself. This is proved by lower MMSE scores, long-term memory deficits [**21**], and decreased verbal fluency [**17**]. These findings are reported constantly in literature, irrespective of the presence of depression [**22**]. 

Cognitive malfunction can be enhanced by the presence of somatic (retinopathy, hypertension, polyneuropathy)[**23**,**24**], and neurological complications (e.g. vascular dementia) [**25**,**26**].

It has also been shown that, during acute hypoglycemia episodes, responses on tests for immediate verbal memory, immediate visual memory, working memory, delayed memory, visual-motor skills, visual-spatial skills, and global cognitive function are all impaired [**27**,**28**]. 

## Quality of life

The main sources for a consistent decrease in the quality of life are the following:

- cognitive impairment itself: patients with MMSE < 23 are constantly worse on measures of self-care and the ability to perform activities of daily living; they report an increased need for personal care and increased rates of hospitalization) [**29**-**32**].

- restrictions in spontaneous decision-making and social implications; typical examples include the following:

- difficulties in leaving home without insulin and food; 

- anxiety caused by the administration of insulin in public;

- daily testing of serum glucose levels; 

- frequent and equally distributed meals; 

- planned physical effort; 

- planned pregnancy;

- food restriction (intake should always be correlated with the insulin dose; consumption of alcohol is restricted); 

- risks of insulin administration itself: hypo- or hyperglycemia; difficulties in learning to distinguish between the types of insulin and their effects, or in learning to adjust doses; lipodystrophy at the injection site.

- sleep disorders, such as insomnia, can be caused by rapid changes in glucose levels during sleep [**33**],or by discomfort/ pain associated with peripheral neuropathy [**34**].

- inability to control the disease: is modulated by factors such as low self-efficacy [**35**], external locus of control, low hardiness, low/ absent coherence, pessimism or unrealistic optimism.

## Psychiatric comorbidity

Depression is a frequent outcome of diabetes. The prevalence of isolated depressive symptoms reaches 30%, whereas the criteria for the major depressive episodes can be met in as high as 10% [**36**].Still, one should also not overlook the risk of masked depression, some patients never reaching the office of a psychiatrist or a psychologist. Middle age patients are more predisposed to depression, possibly on the background of a higher perception of losses and restrictions and/ or a more substantial deterioration of their social roles. Data show that the depression’s expression is mediated by socio-economic status, perceived social support, and gender, especially in women [**37**]. 

Classical psychological mechanisms explaining the onset of depression in diabetic patients include the following:

- the duration of treatment;

- the already deteriorated quality of life (via complications of the disease +/- everyday restrictions [**36**];

- stigma brought by the presence of the disease.

The simple coexistence of depression can increase the care expenses in diabetes, and also mortalityby a factor of 4.5 [**38**]. This is mainly due to poor compliance (equivalent to a bad self-management of the disease and to an increased number of unaddressed complications). Depression and poor compliance can be the key elements for a bad prognosis, because depression promotes low compliance, but also derives from the consequences of a low compliance. 

## Risky behaviors

In diabetes, they can be a problem that potentially affects survival. For example, alcohol consumption has been reported as an important cause of death in patients with type 1 diabetes [**39**].

One of the reasons for risky behaviors that can be often identified is the unrealistic self-assessment of the disease. For example, as many as 40-80% of the people with diabetes underreport their blood sugar levels on at least half of their recordings [**40**]. This can stem from poor education and/ or a poor knowledge of their disease, but it can also reflect a psychological mechanism commonly known as “wishful thinking”. It has a relationship with a distorted sense of self-control, or unrealistic optimism, but it can also be connected to the lack of immediate consequences, if treatment is not properly run. 

A series of theoretical models, generally valid for the explaining attitudes vs. doctor and treatment in chronic diseases, can also be applied in diabetes to understand the deeper roots of non-compliance:

-Health Belief Model(emphasizes the role of the patient’s opinion on treatment gains vs. losses). The imbalance between perceived gains and losses is responsible for up to 2/3 - 3/4 of diabetes patients considering that the prescribed regimen was unsuitable for them [**41**];

-Theory of Reasoned Action(refers to the threshold between what is perceived as a “reasonable” or an “unreasonable” treatment). Influence of family norms and support can be critical for determining the threshold, and, is generally more important in type 1 diabetes, possibly because of its early onset (childhood), when the influence of family is more important [**41**];

- Theory of Planned Behavior(explores what the patient perceives as changeable during the course of the disease). Controllability can be influenced by symptom or regimen changes, but also by preexistent representations of control [**42**];

-Stages of Change Model(placement of the patient in the process of accepting the disease and changing his/ her routines). Typical stages include precontemplation, contemplation, preparation, change, and maintenance or relapse [**43**].

-Leventhal’s Self-Regulation Model(focuses on the role of personal interpretation and core values) [**44**].

## Challenges and limits

In addressing the above-mentioned psychological consequences of diabetes, the therapist can face certain obstacles:

- (Early) diagnosis of cognitive deficitscan be problematic, despite the existence of cognitive impairment tests for adults (e.g. DemTect[**45**], Montréal Cognitive Assessment [**46**]) or imaging methods (fMRI, PET, SPECT, arterial spin labeling MRI [**47**]). The lack of tests availability and/ or instructed personnel can be a serious problem, especially in zones with poor resources for health.

- Complexity of mechanismscan be sometimes puzzling. For example, a vicious circle made up of low adherence/ noxious behavior – deterioration of health – depression – further low adherence/ noxious behavior, or, alternatively, of low self-perceived control – low adherence – even lower self-perceived control can raise the question of the best strategy to follow (in other words “where to begin?”).

- Individual variability(not only somatic, but also psychological) can substantially modify the picture (e.g. hardiness, self-efficacy, strong values, balance gains vs. losses are not easy to detect in a normal Dr-Pt consultation; however, they can substantially influence the outcome).

- Models trying to explain the patient’s attitudescan be problematic.These models are often limited, because they focus only on a group of variables that influence behavior. Furthermore, even when following one model, the patients’ attitudes should be seen in dynamic, thereby requiring the steady involvement of a behavioral specialist. For example, gains vs. losses balance can change sharply; sometimes because of a momentary event (e.g., belief of vulnerability prior to the onset of diabetes can make treatment a reasonable option, BUT belief of vulnerability after complications has occurred despite the treatment and may orient the patient towards alternative medicine).

## Psychological management of diabetes

The responsibility for an effective psychological management of diabetes should be shared between the patient, the physician and, possibly, the psychologist. 

## The patient

Can be trained to apply for several principles of action in the daily confrontation with the challenges of diabetes:

- avoid passive coping mechanisms (denial, projection, repression); address the problem, be active;

- get informed;

- maintain hope and a dose of realistic optimism;

- maintain the collaboration with the doctorand/ or psychologist, even when the disease seems perfectly controllable;

- think on a long term.

## The physician

Should express empathy to the difficulties met by the patient and recognize the early signs of psychological deterioration (e.g. depression, anxiety). The cultivation of empathy generally increases confidence, with a direct positive effect on compliance [**48**], but also on specific behaviors:taking medication as prescribed, planning diet, testing blood glucose, avoiding certain types of food, exercising, monitoring progresses[**49**]. The explanation for this extensive positive outcome is offered by confidence, enhancing the patient’s feeling of being an active participant in the disease management, endowed with a real force of overcoming difficulties.

The physician should also consider prescribing medication for those variables directly involved in modulating compliance (the patient’s representations of gains and losses, group norm, perceived social support, ability vs. desire of control, personal interpretations and strong values, the patient’s progress towards acceptanceof the disease).

## The psychologist

According to patient’s needs, the psychologist can intervene at different levels, from counseling to specific psychotherapeutic techniques. 

Counselingis often constructed under the form of a motivational interview. The key points for a successful counseling are the following:

- expressing empathy(via elements such as active (verbal and nonverbal) listening, expressing comprehension and respect for the patient’s suffering);

- highlighting the differences between present Self and ideal Self(and contribution of the disease to this discrepancy);

- addressing implicit resistance to change (by inviting the patient to take into account an alternative perspective and emphasize its positive consequences); 

- aiming at increasing self-efficacy and confidence, by acknowledging successes and encouraging the desire for future changes [**50**].

Cognitive-behavioral therapy (CBT)represents a step further beyond counseling, as it is more problem-centered. It can be very efficient, as it is generally flexible, focused, and time-limited [**51**].

Phases of CBT include [**52**]:

- specify the problem:avoids the tendency of patients to “catastrophize” it (= to see it as ubiquitous and overwhelming);

- goal setting: the goal should be specific, measurable (how much, how often), action oriented (e.g. to address behavior, rather than physiology) and realistic (not too difficult, so that patients become discouraged, yet difficult enough to give a sense of accomplishment);

- identify barriers to goal attainment (e.g. unrealistic thoughts), counterproductive emotions (e.g. lack of self-esteem), problems with networks (e.g. low social support), problems with resources (e.g. lack of time or money);

- elaborate strategies to overcome barriers:clinicians should ask patients questions, so that theythemselves canformulate ideas and alternatives;

- contracting for change (“behavioral contract”);

- track outcomes (monitor difficulties, reward successes and analyze failures, work on the initial strategy and restructure it, if necessary) and offer continuous support.

## Family Approach to Diabetes Management (FADM)

In this technique, the emphasis falls on helping families changing their offspring’s inadequate behaviors related to diabetes management into more responsible, goal-oriented ones. This is accomplished by modifying the family members’ roles and responsibilities regarding diabetes management. 

FADM is especially effective in teenagers, as it is a directive and intensive approach, based on the concept of mutuality. For example, the adolescent can be given a list of concrete responsibilities regarding the daily management of diabetes; however, these tasks will generally respect the adolescent’s need for autonomy. By discussing abouttheir accomplishment later, in an open manner, the psychologist helps the family and the patient make responsible decisions. This process is mainly based on weighing the benefits and costs associated with the behavioral choices of all family members. This way, the psychologist will address the family and not only a single individual. Consequently, a frequent outcome of a successful FADM is not only a better management of diabetes, but also the development of new, more adaptive ways of functioning for the whole family.

## Challenges and limits

A certain number of potential obstacles should always be considered whenperforming counseling or therapy in diabetic patients:

- lack of addressability to the psychologist,even when symptoms (e.g. depression) manifest;

- lack of patient motivation(especially via unrealistic expectations);

- limits of the therapies themselves(as some require certain abilities from the patient, such as insight, or the genuine intention for a lifestyle change).

Despite these limits, the use of human and conceptual resources offered by Psychology can be undoubtedly considered an essential part of a better and modern management of diabetic patients. 
